# Follicular Dendritic Cell Sarcoma With Co-Expression of CD4 and CD30 Mimics Anaplastic Large Cell Lymphoma

**DOI:** 10.3389/fonc.2020.00876

**Published:** 2020-05-29

**Authors:** Hui Liu, Chenxi Xiang, Mei Wu, Shimin Hu

**Affiliations:** ^1^Department of Pathology, Xuzhou Medical University, Xuzhou, China; ^2^Department of Pathology, The Affiliated Hospital of Xuzhou Medical University, Xuzhou, China; ^3^Department of Hematopathology, The University of Texas MD Anderson Cancer Center, Houston, TX, United States

**Keywords:** Follicular dendritic cell sarcoma, anaplastic large cell lymphoma, pathologic spectrum, CD30, CD4

## Abstract

Follicular dendritic cell sarcoma (FDCS) is a low-grade malignant neoplasm that tends to be under-recognized owing to its rarity and wide pathologic spectrum. Knowledge of the atypical morphology and immunophenotype of FDCS is critical to avoid misdiagnosis. Here we presented a case of extranodal FDCS with an unusual morphology and a previously unreported immunophenotype leading to misdiagnosis. A 32-years-old man presented with a tonsilar mass that showed epithelioid cells in nested and alveolar patterns. Immunohistochemistry study revealed that the tumor cells were positive for CD4 and CD30, and were negative for cytokeratin, CD3, CD20, CD68, CD163, lysozyme, ALK, S-100, and desmin. Multiple outside expert consultations rendered a consensus diagnosis of ALK-negative anaplastic large cell lymphoma (ALCL). The patient received multiple lines of chemotherapy and radiotherapy. However, the residual tumor progressively enlarged eight months later and a more complex morphology was presented in the re-excised tumor: including spindle cells with vesicular nuclei and nuclear pseudoinclusions in fascicles or a whorled pattern, and plump ovoid cells arranged in meningioma-like whorls as well as epithelioid tumor cells similar to the initial biopsy. All these three components were positive for CD4, CD21, CD23, and CD35. The diagnosis was revised to FDCS after a positive immunostaining for CD21, CD23, and CD35 on the initial specimen was confirmed retrospectively. A literature review identified 57 cases of FDCS published from 2009 through 2019, and 13 (22.8%) of them were misdiagnosed at initial presentation. Among these misdiagnosed cases, all except one case were extranodal, and the incorrect initial diagnosis was mostly location-related. These cases expand the pathologic spectrum of FDCS, and further emphasize the necessity for pathologists to stay alert for this rare entity, bringing FDCS into the differentials for any spindle cell tumors, undifferentiated epithelioid cell tumors, and ALCL to avoid misdiagnosis.

## Introduction

Follicular dendritic cell sarcoma (FDCS) is an uncommon neoplasm involving lymph nodes or extranodal sites. It occurs in adult patients without a gender predisposition. Histologically, FDCS consists mainly of spindle and ovoid cells growing in fascicles, sheets, whorls, or a storiform pattern with small lymphocytes dispersing throughout the tumor. The tumor cells typically express FDC markers, including CD21, CD23, and CD35. D2-40, CXCL13, and clusterin are usually positive though not specific. These distinct pathologic features make the diagnosis of nodal FDCS relatively straightforward, given that not many types of spindle cell tumors occur in lymph nodes.

When presenting as an extranodal neoplasm, however, spindle cell tumors do not always cause concern for FDCS, due to its rarity compared with spindle cell sarcoma or carcinoma. Moreover, the broad spectrum of morphology makes the diagnosis of FDCS more challenging. The reported rate of misdiagnosis of FDCS at extranodal sites is up to 58% (1), supporting that FDCS has obviously been underrecognizied.

We reported herein a unique case of tonsillar FDCS with an unusual morphology and a previously unreported immunophenotype, leading to the misdiagnosis as ALK-negative anaplastic large cell lymphoma (ALCL) initially. We further reviewed cases of FDCS published from 2009 though 2019. The rate of misdiagnosis of FDCS, especially extronodal cases, was analyzed.

## Case Report

**Figure 1:**
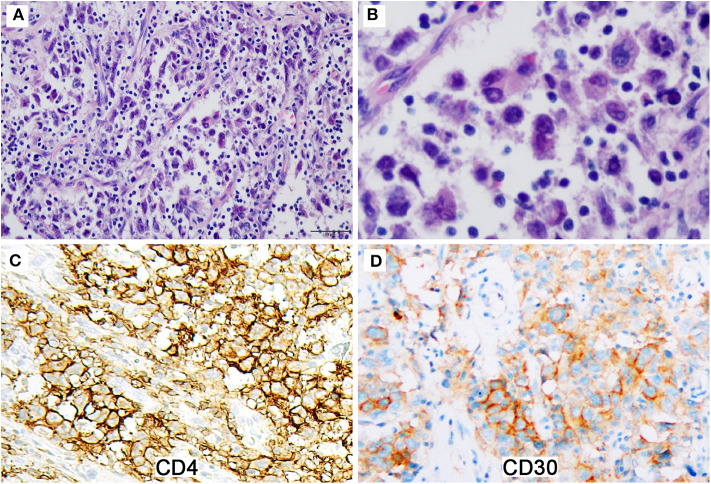
The initial excisional specimen shows epithelioid tumor cells with co-expression of CD4 and CD30. **(A)** Epithelioid tumor cells arrange in nested and alveolar patterns with small lymphocytes scattered through the lesion (Magnification: 200 ×). **(B)** The tumor cells are large to pleomorphic with slightly irregular nuclei and abundant eosinophilic cytoplasm (Magnification: 400 ×). **(C,D)** The tumor cells are strongly positive for CD4 **(C)** and moderately positive for CD30 **(D)** (Magnification: 400 ×).

A 32-years-old man presented with a two-month history of hoarse voice and difficulty to swallow. Computed tomography scan revealed a 3.1 × 2.0 × 2.0 cm isolated right tonsillar mass without lymphadenopathy or any additional lesions. An excisional biopsy demonstrated a tonsil extensively replaced by epithelioid cells in nested and alveolar patterns (Figures 1A,B). Small lymphocytes were scattered through the lesion. The tumor cells were large to pleomorphic with slightly irregular vesicular nuclei, small nucleoli, and abundant eosinophilic cytoplasm. Multinucleated giant cells were scattered. The tumor cells were positive for CD4 and CD30 (Figures 1C,D), and negative for ALK, CD2, CD3, CD20, CD68, CD163, lysozyme, cytokeratin, S100, and desmin. A consensus diagnosis of ALK-negative ALCL was rendered upon multiple outside expert consultations. The patient received multiple lines of chemotherapy including EPOCH (etoposide, prednisone, vincristine, cyclophosphamide, and doxorubicin hydrochloride), ESHAP (etoposide, methylprednisone, cytarabine and cisplatin), and GDP (gemcitabine, dexamethasone and cisplatin) as well as radiotherapy.

**Figure 2:**
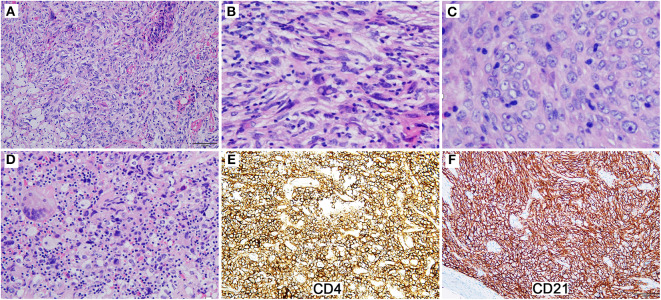
The reexcised tumor exhibits multiple morphologic components. **(A)** Pleomorphic spindle cells grow in fascicles or a storiform pattern (Magnification: 200 ×). **(B)** The tumor cells show nuclear pseudoinclusions (Magnification: 400 ×). **(C)** Relative monotonous plump spindle cells arrange in meningioma-like whorls (Magnification: 400 ×). **(D)** Clusters of foamy cells and inflammatory cells with foreign body-giant cell reaction are presented (Magnification: 200 ×). **(E,F)** The tumor cells are diffusely and strongly positive for CD4 **(E)** and CD21 **(F)** (Magnification: 200 ×).

Eight months later, the patient presented with nosebleed. Computed tomography scan showed a 2.5 × 2.1 × 2.1 cm isolated mass in the right oropharyngeal area. Histologic examination of the excised mass revealed a tumor exhibiting multiple morphologic components with numerous pleomorphic spindle cells growing in fascicles or a storiform pattern being the major component (Figures 2A,B). Nuclear pseudoinclusions were easily seen in these cells. Relative monotonous plump spindle or ovoid cells arranged in meningioma-like whorls (Figure 2C) and epithelioid tumor cells seen in the initial biopsy were also presented focally. Clusters of foamy cells and cholesterol-like crystal deposit with foreign body-giant cell reaction were seen peripherally (Figure 2D). The tumor cells were positive for CD4, CD21, CD23, and CD35 (Figures 2E,F), and negative for ALK, CD3, CD20, CD30, CD45, cytokeratin, S-100, HMB45, desmin, and EBER. Immunostains for CD21, CD23, and CD35 performed retrospectively on the initial specimen were positive in the tumor cells. TCR rearrangement analyses were performed at an outside consultation institution, and no monoclonal TCR gamma or TCR beta rearrangement was detected. The diagnosis was revised to FDCS. The patient was on observation and was well 32 months after the second surgery.

## Literature Review

A PubMed review of FDCS cases reported in the English literature from 2009 through 2019 was conducted. Fifty-seven cases of FDCS were identified, including 11 (19.3%) nodal cases and 46 (80.7%) extranodal cases. The most common primary extranodal sites involved by FDCS were the abdominal cavity (21/57, 36.8%), followed by head and neck region (12/57, 21.1%), and less frequently, mediastinum, bladder, and other organs (breast, renal, and brain) (data not shown). A total of 13 (13/57, 22.8%) cases were misdiagnosed at initial presentation. Ten cases of FDCS misdiagnosed as other types of neoplasm: carcinoma (*n* = 4), gastrointestinal stromal tumor (*n* = 2), classic Hodgkin lymphoma (*n* = 2), and malignancy not classified (*n* = 2), respectively. Three cases of epithelial tumor were misdiagnosed as FDCS: urothelial carcinoma, sarcomatoid carcinoma, and juxtaglomerular cell tumor each. Twelve of 13 cases were extranoal lesions. The misdiagnosis rate of extranodal FDCS (12/46, 26.1%) was higher than that of nodal counterpart (1/11, 9.1%), although no statistical significance was reached. The initial incorrect diagnosis was strongly related to the involved anatomic locations (Table 1). For example, tumors in colon, bladder, and tonsil were more frequently to be misdiagnosed as gastrointestinal stromal tumor or poorly differentiated carcinoma, wheras mediastinal lesions were more likely to be recognized as classic Hodgkin lymphoma.

**Table 1:** Information of 13 misdiagnosed FDCS cases retrieved from PubMed between 2009 and 2019.

**No**.	**Author**	**Age**	**Gender**	**Tumor site**	**Initial diagnosis**	**Final diagnosis**
1	Gupta et al. (2)	28	M	Colon	GIST	FDCS
2		63	M	Colon	GIST	FDCS
3	Wu et al. (3)	52	F	Mediastinum	CHL(needle biopsy)	FDCS
4		17	F	Mediastinum	Lymphoma	FDCS
5	Duan et al. (1)	63	F	Urinary bladder	A malignant tumor, not further classifiable (preoperational biopsy)	FDCS
6	Zaya et al. (4)	78	F	Vagina	Squamous cell carcinoma	FDCS
7	Lu et al. (5)	59	M	Tonsil	A poorly-differentiated malignant tumor	FDCS
8		46	M	Tongue	Squamous cell carcinoma	FDCS
9	Kapucuoglu et al. (6)	31	F	Breast	Poorly differentiated carcinoma	FDCS
10	Wu et al. (7)	45	M	Supraclavicular fossa node	Poorly differentiated carcinoma	FDCS
11	Sun et al. (8)	73	M	Urinary bladder	FDCS	Urothelial carcinoma with FDCS
12		41	M	Neck node	FDCS (expert 1); Sarcomatoid carcinoma (expert 2)	Sarcomatoid carcinoma
13	Lopez-Hisijos et al. (9)	29	F	Kidney	FDCS	Juxtaglomerular Cell Tumor (JCT)

## Discussion

FDCS is a neoplastic proliferation of spindle to ovoid follicular dendritic cells characteristically arranged in fascicles, storiform arrays, or whorled patterns. FDC markers, including CD21, CD23, and CD35, are typically expressed by the tumor cells. These markers, together with clusterin, D2-40, and EGFR, which are also sensitive markers for FDCS, facilitate the diagnosis when FDCS is raised among the differential diagnoses. However, cases of FDCS with atypical pathologic features were reported in the literature. And the wide spectrum of morphology makes the diagnosis challenging.

Morphologically, the tumor cells of FDCS range from being spindle or oval to epithelioid (10), pleomorphic or anaplastic cells (11, 12), multinucleated (10), or Reed-Sternberg-like. The architectures also vary in FDCS, including the typical fascicles, whorls, and storiformed arrays, as well as angiomatoid or puzzle-like pattern (13). Unusual background, like myxoid or densely hyalined, may display (14). Regardless of the type of cytomorphology or growth pattern, the sprinkling of small lymphocytes within the tumor or clustered around vessels serves as a helpful clue to FDCS. FDC specific markers, like CD21, CD23, and CD35, are ready to establish the right diagnosis once FDCS is included in the differential diagnoses. Clusterin, D2-40, CXCL13 are also positive mostly (15). However, the neoplastic FDCS cells express EMA (16, 17), S-100 (17), CD68 (12), and even cytokeratin (12) occasionally, leading to a misdiagnosis toward carcinoma, miningeoma, melanoma, or spindle cell sarcoma, etc., particularly, when FDCS displays atypical morphologic features and FDC markers are not stained.

Atypical epithelioid morphology of FDCS is rare, and reported in literatures mostly as individual cases (10), and usually co-exists with other classical FDCS morphology, which assists pathologists in diagnosis. FDCS mimicking ALCL morphologically is much rarer. Wu et al. (2) recently reported an unusual case of FDCS in the left supraclavicular node with mixed morphologic patterns resembling desmoplastic infiltrative carcinoma, hemangiopericytoma, classical Hodgkin's lymphoma, and ALCL, along with a component of FDCS with classical morphology. Although the atypical round or polygonal large tumor cells arranged in a nested or patchy pattern closely resembling ALCL, these cells did not express CD30 but CD21, CD23, and CD35, guiding the pathologists to the diagnosis of FDCS.

The case we reported here displayed epithelioid cellular features and a nested or alveolar growth pattern, raising suspicion of poorly differentiated carcinoma initially. Immunohistochemistry revealed that the neoplastic cells were negative for epithelial, T- or B-lineage, and melanoma markers, but positive for CD4 and CD30, although the staining pattern of CD30 was not homogenously strong. Aberrant CD4 expression by FDCS was extremely rare (18). CD30 expression, particularly co-expression with CD4, in FDCS was not reported so far to our knowledge (19). The co-expression of CD4 and CD30 combined with the unusual morphology raised the diagnosis of ALCL, particularly, when no other specific markers were performed. No suspicion about the diagnosis of FDCS was raised until the observation of a component with typical FDCS morphology in the second excisional specimen. Positive immunohistochemical staining for CD21, CD23, CD35 on the initial specimen confirmed the diagnosis of FDCS. However, CD30 immunostaining was negative on the reexcised tumor cells. It is not known whether it is related to treatment. From a retrospective view, the diffuse but heterogeneous expression of CD30 on the initial biopsy specimen, which is not typical for ALCL, should have made us thinking twice.

Although FDCS most often occur *de novo*, occasionally it may arise in the setting of hyaline-vascular Castleman disease (20). The patient we reported here did not have any significant past medical history including Castleman disease. Interestingly, Walters et al. reported that nearly half of FDCS cases demonstrated indolent T-lymphoblastic proliferation, and some of these patients developed autoimmune diseases (21). However, we did not observe TdT-positive T-cells in both specimens from our patient, and no evidence was found to suggest an autoimmune disease in our patient.

Although increasingly recognized by pathologists in recent years, FDCS appears susceptible to diagnostic pitfalls because of its rarity and wide spectrum of pathologic features, especially when it comes to extranodal disease. We retrieved 57 FDCS cases in PubMed published from 2009 through 2019, and found that extranodal FDCS cases were more frequently reported than nodal disease (80.7 vs. 19.3%), and the most frequently involved extranodal location was abdominal cavity, followed by head and neck region. Also, we found that the misdiagnosis rate of extranodal FDCS cases was slightly lower than previously reported (~58%) (1), but still higher than that of nodal diseases (26.1 vs. 9.1%), although the difference was not significant statistically. Thirteen cases were misdiagnosed: either FDCS as other tumor types or other tumor types as FDCS. The initial misdiagnosis seems related to the involved anatomic location. This result suggests that FDCS tends to be underrecognized by pathologists when presenting as extranodal lesions and a differential of FDCS not raised.

## Conclusion

The case we presented here, with peculiar immunophenotypic and morphologic features, expands the pathologic spectrum of FDCS, and further emphasizes the necessity for pathologists to stay alert for this rare entity, bringing FDCS into the differentials for any spindle cell tumors, undifferentiated epithelioid cell tumors, and ALCL, to avoid misdiagnosis.

## Data Availability Statement

The raw data supporting the conclusions of this article will be made available by the authors, without undue reservation, to any qualified researcher.

## Ethics Statement

The studies involving human participants were reviewed and approved by Ethics Committee of The Affiliated Hospital of Xuzhou Medical University. Written informed consent for participation was not required for this study in accordance with the national legislation and the institutional requirements.

## Author Contributions

HL designed the study and wrote the manuscript. CX conducted PubMed search and analyzed the data. MW wrote the first draft of the manuscript. SH revised the manuscript.

## Conflict of Interest

The authors declare that the research was conducted in the absence of any commercial or financial relationships that could be construed as a potential conflict of interest.
